# Front-of-Pack Nutrition Labelling Schemes: Where Are We Now?

**DOI:** 10.3390/nu15184001

**Published:** 2023-09-15

**Authors:** Antonis Zampelas

**Affiliations:** 1 Laboratory of Dietetics and Quality of Life, Department of Food Science and Human Nutrition, Agricultural University of Athens, 11588 Athens, Greece; azampelas@aua.gr; 2 Hellenic Food Authority, Leoforos Kifissias 124 & Iatridou 2, 11526 Athens, Greece

The development of nutritional policies plays a vital role in health promotion. With respect to nutritional labeling, the food industry and food authorities have proposed various schemes, all with their advantages and disadvantages. Relatively recently, front-of-pack labeling has received attention as a potential nutritional policy that could contribute to the reduction of nutrition-related diseases. The goal is to help consumers to make informed decisions on the healthiness of the products they purchase. In this Special Issue of *Nutrients* on “Front of Pack Nutrition Labelling” (FOPNL), thirteen interesting studies aim to shed additional light on the challenges that law-makers have been facing in order to develop policies that can inform consumers to make better choices and subsequently change their nutrition behavior and improve their health.

In two of the studies published in this issue, various FOPNL schemes were compared and Nutri-Score seemed to be ranked more highly than the others [1,2]. In particular, in the first of these studies, Francisco Goiana-da-Silva et al. (2021) compared five FOPNL schemes (Health Star Rating (HSR), Multiple Traffic Lights (MTLs), Nutri-Score, Reference Intakes (RIs), or Warning Symbol (WS)) [1]. The results of this study showed that although all these FOPNLs led to a higher percentage of correct responses compared to the no-label approach, Nutri-Score produced the greatest improvements. In the second study, which was carried out in Greece, where the Greek Government officially supports the implementation of a descriptive but not evaluative type of FOPNL, Kontopoulou et al. (2022) investigated the understanding and perceptions of Greek consumers in response to five different FONLs: MTL, HSR, Guideline Daily Amounts (GDAs), WS, and Nutri-Score [2]. The results of this study also showed that the use of Nutri-Score led to greater improvements when compared to the GDA for Greek consumers, and more importantly, the authors concluded that Nutri-Score helped Greek consumers to rank foods more efficiently according to their nutritional value. In another study, which was carried out in France, Ducrot P et al. (2022) assessed Nutri-Score awareness, perception, and self-reported impact and their determinants on food choices in French adolescents [3]. In this population, almost all the adolescents reported awareness of Nutri-Score, more than 9 out of 10 respondents considered the logo easy to understand and easy to identify on food packages, and approximately half of them self-reported that the label had already impacted their food choices. Regarding determinants, the use of Nutri-Score by the parents was the most profound determinant criterion for its use by the participants.

Kuhne et al. (2022) used a newly developed swipe task to investigate whether the type of label used (summary vs. nutrient-specific) had differential effects on different operationalizations of the “healthier choice” measure (e.g., calories and sugar) [4]. Participants were randomly allocated to one of five groups: two summary label groups (Nutri-Score and Healthy Food Label (HFL)), two nutrient (sugar)-specific label groups (manga and comics), or a control group without a label. The results of this study showed that more “healthy” products were purchased in the label conditions than in the control condition. However, interestingly, it was also argued that the increase in the purchase of green-labeled healthy products (in label conditions) could “compensate” for the purchase of red-labeled unhealthy products and consequently could lead to an increase in total energy intake.

In their study, Silva et al. (2022) pointed out that on the one hand, Portuguese consumers have the habit of reading food labels, recognizing its importance, but on the other hand, they do not understand all the information contained in it [5]. It is also noteworthy that consumers found it easier to understand FOPNLs and especially those presented through symbols/colors. These results are in agreement with the results of the study of Sajdakowska et al. [6] which indicated the obvious: that consumers are discouraged from reading label information when a large amount of information is presented to them, and they are generally reluctant to take an interest in label information because, at least in part, the amount and complexity of information currently appearing on food labels discourages them from reading it.

In order to assist consumers, Fuchs et al. (2022) developed a Chrome web browser extension for a real online supermarket and evaluated the effect of this digital food label intervention (i.e., displaying the Nutri-Score next to visible products) on the nutritional quality of individuals’ weekly grocery shopping in a randomized controlled laboratory trial [7]. Compared to the control group, individuals exposed to the intervention chose products with a higher nutritional quality and users with low food literacy also benefited from the digital FOPNL.

The purpose of another study by Campos-Nonato et al. (2022) was to compare the perception and understanding of GDAs and WS [8]. When comparing the same products with different labels, the participants reported that it would be unlikely/very unlikely that they would consume products packaged with the WL compared to those with GDAs. In addition, their perception was that the quantities of packaged products they should consume was small or very small when they used the WS compared to GDAs and they were more confident about choosing healthy products when using the WS compared to the GDA.

In another study by Cristian Adasme-Berríos et al. (2002), it was reported that Nutrition Warnings (NWs) increase the avoidance of processed foods [9]. The message provided by NWs had a mediating effect between the intention to avoid processed food and eating motivation, but showed no such effect on nutritional knowledge. The findings of this study suggested that NWs are able to turn negative eating motivation into positive eating motivation to avoid processed foods. However, it has to be noted that there are various processed foods of high nutritional value and consequently all types of FOPNLs should take this into account to avoid consumer misinformation.

In a South African group of participants, Melvi Todd et al. (2022) compared FOPNLs (Control, HSR, GDA, with a ‘less healthy’ nutritional profile, endorsement logo/low Glycemic Index claim, low HSR, GDA with a ‘healthy’ nutritional profile, and WS) as a quick assessment tool [10]. The results of this study showed that in terms of helping consumers identify less healthy products, the effect sizes were most prominent for WS and low HSR.

Wei et al. (2022) investigated the understanding and use of nutrition labels of prepackaged food by university students in four different fields of study in Chongqing, China [11]. It was striking that only 21.3% of the students had a good understanding of the nutrition labels of prepackaged food, with medical students having the greatest understanding.

In another study from Greece, Katsouri et al. (2022) investigated labelling in pre-packed cheeses. In total, 158 prepacked products belonging to 19 “quality label” cheeses were identified in the Greek market [12]. All labelling indications, nutritional information, claims, and other labelling data were recorded and analyzed in relation to their compliance against European food law requirements. The results of the analysis showed that for only 6 of the 19 cheeses, all products fully complied with EU labelling legislation and that among the 14 mandatory labelling requirements, the lowest overall compliance was observed for allergen declaration (65%). It is therefore obvious that national authorities should also focus not only on what is written on labels but also on whether what is written is true.

In an interesting study by George Marakis (2021) et al. it was reported that most participating households in Greece used olive oil produced by themselves or by their extended family or friends (60.3%), which shows that policy makers should not only focus on FOPNLs but also on other consumer misconceptions [13].

Taking into consideration the main results of the papers published in this Special Issue of *Nutrients*, it could be suggested that consumers prefer simple schemes, preferably with colors. In addition, the awareness and the magnitude of the use of FOPNLs vary among countries and the education levels of the consumers. Therefore, when a scheme is decided, significant education campaigns must also be designed and efficiently implemented, primarily by National Authorities. What also has to be emphasized is that the consumer should not receive the impression that healthier foods can be consumed freely, because then they will contribute significantly to the total energy intake and consequently may lead to an increase in body weight. Therefore, all these issues should be taken into account when incorporated into the National Nutrition Guidelines.
